# Supplementary material for: Emotional Response Inhibition: A Shared Neurocognitive Deficit in Eating Disorder Symptoms and Nonsuicidal Self-Injury
Source: Brain Sci. 2020 Feb 15;10(2):104. doi: 10.3390/brainsci10020104 (PMC7071419; doi:10.3390/brainsci10020104)
Supplement: Supplementary file 1 [file brainsci-10-00104-s001.pdf]

Supplementary Material: Emotional Response Inhibition: A Shared Neurocognitive Deficit in Eating Disorder Symptoms and Nonsuicidal Self-Injury

Table S1. ZINB regression analyses predicting past-month overeating frequency (N = 102)

	**EDE-Q Overeating**			
	**Logistic Model**		**Count Model**	
**Step 1.**	***B (SE)***	**IRR**	***B (SE)***	**IRR**
**Sex**	0.56 (1.28)	1.75	0.53 (0.56)	1.69
**Gender/Orientation**	0.71 (1.00)	2.03	0.66 (0.59)	1.93
**Race**	0.28 (0.66)	1.32	-0.50 (0.34)	0.61
**NSSI History**	-0.23 (0.98)	0.79	0.10 (0.50)	1.10
**ESST Accuracy**	-0.05 (0.03)	0.95	**-0.06 (0.21)****	**0.95**
**Step 1:**	χ^2(10, 89) = 16.47, Log-likelihood: -195.49			
**Step 2.**	***B (SE)***	**IRR**	***B (SE)***	**IRR**
**Negative Urgency**	**-0.09 (0.04)***	**0.92**	**0.05 (0.02)****	**1.06**
**NEAT**[a]	0.00 (0.03)	1.00	**0.03 (0.01)***	**1.03**
**Step 2:**	χ^2(2, 87) = **19.44*****, Log-likelihood: **-186.08**			
**Step 2 model comparison:**	χ^2(0, 15) = **11.36*****, Log-likelihood: **-191.76**[a]			
**Step 3.**	***B (SE)***	**IRR**	***B (SE)***	**IRR**
**Negative Urgency**	**-0.08 (0.04)***	**0.92***	**0.05 (0.02)****	**1.05**
**NEAT**	0.00 (0.03)	1.00	**0.02 (0.01)***	**1.02**
**Step 3:**	χ^2(2, 85) = 5.28, Log-likelihood: -183.52			
**Full Model:**	χ^2(14, 85) = **45.78*****, AIC: 401.04			

Note. IRR = Incident Risk Ratio; AIC = Akaike's Information Criterion. [a]Model statistics with NEAT entered at Step 2 (prior to negative urgency); model comparison evaluated using likelihood ratio testing. All other chi-square values obtained at each step via Wald tests; full model chi-square derived from comparison against null (constant-only) model. *p < .05; **p < .01; ***p < .001.

Table S2. ZINB regression analyses predicting past-month loss-of-control (LOC) eating frequency (*N* = 102)

	EDE-Q LOC Eating			
	Logistic Model		Count Model	
**Step 1.**	*B (SE)*	IRR	*B (SE)*	IRR
**Sex**	0.54 (1.13)	1.71	**1.44 (0.69)***	**4.24**
**Gender/Orientation**	0.96 (1.02)	2.62	**1.43 (0.69)***	**4.17**
**Race**	1.30 (0.70)	3.66	0.28 (0.49)	1.33
**NSSI History**	-1.23 (0.94)	0.29	-0.51 (0.56)	0.60
**ESST Accuracy**	0.08 (0.06)	1.08	0.03 (0.02)	1.03
**Step 1:**	χ^2(10, 89) = **22.74***, Log-likelihood: **-139.26**			
**Step 2.**	*B (SE)*	IRR	*B (SE)*	IRR
**Negative Urgency**	-0.12 (0.07)	0.89	**0.06 (0.03)***	**1.06**
**NEAT[a]**	-0.05 (0.03)	0.95	**0.03 (0.02)***	**1.03**
**Step 2:**	χ^2(2, 87) = **12.06****, Log-likelihood: **-131.65**			
**Step 2 model comparison:**	χ^2(0, 15) = **4.54*****, Log-likelihood: **-133.92[a]**			
**Step 3.**	*B (SE)*	IRR	*B (SE)*	IRR
**Negative Urgency**	-0.05 (0.04)	0.96	**0.06 (0.02)****	**1.07**
**NEAT**	-0.03 (0.02)	0.98	**0.03 (0.01)***	**1.03**
**Step 3:**	χ^2(2, 85) = **9.86****, Log-likelihood: **-129.62**			
**Full Model:**	χ^2(14, 85) = **47.51*****, AIC: 293.25			

Note. IRR = Incident Risk Ratio; AIC = Akaike's Information Criterion. [a]Model statistics with NEAT entered at Step 2 (prior to negative urgency); model comparison evaluated using likelihood ratio testing. All other chi-square values obtained at each step via Wald tests; full model chi-square derived from comparison against null (constant-only) model. *$p < .05$; **$p < .01$; ***$p < .001$.

Table S3. ZINB regression analyses predicting binge eating frequency (*N* = 102)

	EDE-Q Binge Eating Days			
	Logistic Model		Count Model	
**Step 1.**	*B (SE)*	IRR	*B (SE)*	IRR
**Sex**	1.59 (1.50)	5.10	**1.44 (0.58)***	**4.22**
**Gender/Orientation**	0.75 (0.84)	1.88	0.99 (0.54)	2.51
**Race/Ethnicity**	0.99 (0.72)	2.57	-0.77 (0.51)	0.46
**NSSI History**	-0.99 (0.79)	0.43	-0.34 (0.50)	0.71
**ESST Accuracy**	0.12 (0.07)	1.13	**0.05 (0.02)***	**1.06**
**Step 1:**	χ^2(10, 89) = **21.34***, Log-likelihood: **-131.74**			
**Step 2.**	*B (SE)*	IRR	*B (SE)*	IRR
**Negative Urgency**	**-0.12 (0.05)***	**0.89**	**0.07 (0.02)****	**1.07**
**NEAT[a]**	-0.08 (0.03)	0.92	**0.05 (0.02)****	**1.05**
**Step 2:**	χ^2(2, 87) = **18.93*****, Log-likelihood: **-121.60**			
**Step 2 model comparison:**	χ^2(0, 15) = **1.36*****, Log-likelihood: **-122.28[a]**			
**Step 3.**	*B (SE)*	IRR	*B (SE)*	IRR
**Negative Urgency**	-2.55 (2.44)	0.08	**0.07 (0.02)*****	**1.08**
**NEAT**	-6.38 (6.07)	0.00	0.02 (0.02)	1.02
**Step 3:**	χ^2(2, 85) = 2.46, Log-likelihood: -106.11			
**Full Model:**	χ^2(14, 85) = **41.93*****, AIC: 246.22			

Note. IRR = Incident Risk Ratio; AIC = Akaike's Information Criterion. [a]Model statistics with NEAT entered at Step 2 (prior to negative urgency); model comparison evaluated using likelihood ratio testing. All other chi-square values obtained at each step via Wald tests; full model chi-square derived from comparison against null (constant-only) model. *$p < .05$; **$p < .01$; ***$p < .001$.

Supplementary Table 4. ZINB regression analyses predicting frequency of past-month compensatory behaviors (*N* = 102)

	EDE-Q Compensatory Behaviors			
	Logistic Model		Count Model	
**Step 1.**	***B (SE)***	**IRR**	***B (SE)***	**IRR**
**Sex**	14.27 (223.19)	158.15[e4]	**2.64 (0.43)*** **	**13.96**
**Gender/Orientation**	1.75 (1.04)	5.75	**2.31 (0.41)*** **	**10.09**
**Race**	0.01 (0.57)	1.00	**0.67 (0.32)***	**1.96**
**NSSI History**	-1.86 (0.97)	0.16	**-1.14 (0.36)** **	**0.32**
**ESST Accuracy**	0.05 (0.04)	1.05	0.02 (0.01)	1.02
**Step 1:**	χ^2(10, 89) = **54.29*****, Log-likelihood: **-153.51**			
**Step 2.**	***B (SE)***	**IRR**	***B (SE)***	**IRR**
**Negative Urgency**	-0.05 (0.04)	0.95	0.00 (0.02)	1.00
**NEAT[a]**	**-0.08 (0.04)***	**0.92**	-0.02 (0.01)	0.98
**Step 2:**	χ^2(2, 87) = 1.54, Log-likelihood: -152.70			
**Step 2 model comparison:**	χ^2(0, 15) = **8.97*****, Log-likelihood: **-148.22[a]**			
**Step 3.**	***B (SE)***	**IRR**	***B (SE)***	**IRR**
**Negative Urgency**	-0.06 (0.05)	0.94	0.01 (0.02)	1.00
**NEAT**	**-0.09 (0.04)***	**0.92**	-0.03 (0.02)	0.98
**Step 3:**	χ^2(2, 85) = **7.98***, Log-likelihood: **-147.33**			
**Full Model:**	χ^2(14, 85) = **57.65*****, AIC: 328.65			

Note. IRR = Incident Risk Ratio; AIC = Akaike's Information Criterion. [a]Model statistics with NEAT entered at Step 2 (prior to negative urgency); model comparison evaluated using likelihood ratio testing. All other chi-square values obtained at each step via Wald tests; full model chi-square derived from comparison against null (constant-only) model. *$p < .05$; **$p < .01$; ***$p < .001$.

Supplementary Results

Alternative model for EDE-Q Weight Concerns (see Footnote 2 in the main text)

We ran an exploratory alternative model for EDE-Q Weight Scores, in which we entered NEAT on Step 2 ahead of Negative Urgency (entered on Step 3). In this model, NEAT had a main effect on EDE-Q Weight Concerns, B = 0.02, SE = 0.01, $F_{change}(1, 95) = 4.95$, $p = 0.28$, $R^2_{change} = .04$. NEAT's effect on this subscale (B = 0.02, SE = 0.01, $\beta = 0.17$, $p = 0.64$) was no longer evident when Negative Urgency was included in the final step (B = 0.06, SE = 0.00, $\beta = 0.30$, $p = 0.003$), confirming that Negative Urgency had additional predictive utility beyond the effect of NEAT.
